# Combined surgical treatment of esophageal cancer and coronary heart diseases in elderly patients

**DOI:** 10.1186/s12957-018-1512-5

**Published:** 2018-10-24

**Authors:** Weiran Zhang, Ban Liu, Yue Zhou, Feng Wang, Chang Gu, Qi Wang, Xiaofang Wang, Yangyang Zhang

**Affiliations:** 1 Department of Cardiothoracic Surgery, BenQ Hospital, Affiliated Hospital of Nanjing Medical University, Nanjing, China; 2 Department of Cardiology, Shanghai Tenth People’s Hospital, Tongji University School of Medicine, Shanghai, China; 3 Department of Cardiothoracic Surgery, First Affiliated Hospital of Nanjing Medical University, Nanjing, China; 4 Department of Gastroenterology, Shanghai Tenth People’s Hospital, Tongji University School of Medicine, Shanghai, China; 5 Department of Thoracic Surgery, Shanghai Chest Hospital, Shanghai Jiao Tong University, Shanghai, China; 6 The Clinical Medical Department of Nanjing Medical University, Nanjing, China; 7 Department of Cardiovascular Surgery, Shanghai East Hospital, Tongji University School of Medicine, 150 Jimo Road, Shanghai, 200120 China

**Keywords:** Simultaneous procedures, Esophagectomy, Off-pump, Coronary artery bypass grafting, The elderly

## Abstract

**Objective:**

The co-incidence of esophageal cancer and coronary heart disease (CHD) is increasing in elderly patients. This study was carried out to analyze the efficiency and safety of simultaneous esophagectomy and cardiac surgery in a selected group of elderly patients.

**Methods:**

Prospective database for coexistency of severe CHD and esophageal or esophageal-gastric junction cancer was firstly reviewed. Twenty-two patients undergoing combined surgical interventions, including first beating-heart coronary artery bypass grafting (off-pump CABG) and then esophagectomy, were involved as group A. Then, 44 patients undergoing isolated esophagectomy were selected as group B using the propensity score matching method. Data including clinic pathological characteristics and postoperative outcomes were investigated. Kaplan–Meier analysis was used.

**Results:**

The surgical procedure was performed through left lateral thoracotomy in all patients, except one patient in group A who received median sternotomy and left lateral thoracotomy. The operation time and blood loss were both more in group A, as a result of two operations performed at one session. Patients in both groups were followed up from 1.3 to 78.3 months. No significant between-group was found in overall survival or relapse-free survival.

**Conclusion:**

The risk of simultaneous esophagectomy and cardiac surgery is not high. Despite certain differences in clinical indicators between groups, the safety of simultaneous procedures in group A is evident.

**Trial registration:**

ChiCTR 1800014498. Registered 17 January 2018

**Electronic supplementary material:**

The online version of this article (10.1186/s12957-018-1512-5) contains supplementary material, which is available to authorized users.

## Introduction

China has begun to enter an aging society, while age is the main risk factor for coronary heart disease (CHD) and cancers. CHD is the leading cause of death in the world, accounting for almost one third of all global deaths, and is the secondary cause of death in China [1, 2]. Esophageal cancer, a most common cancer worldwide, is the leading cause of death among all cancers and mostly (over 80%) attacks developing countries [3]. In China, the incidence of esophageal cancer is about twofold higher than that in the world [4].

Since early-staged esophageal cancer is asymptomatic, most patients were diagnosed at the advanced stage, leading to poor survival [4]. Despite significant development in multimodality treatment of esophageal cancer, including chemotherapy, radiation, and targeted therapies, surgical resection is still the most effective means to achieve long-term disease-free survival in patients with early-staged esophageal cancer. Coexistence of malignant disease (e.g., esophageal cancer) and CHD is common and is expected to increase due to diagnostic improvement and the aging population. However, it is very difficult to decide how to treat CHD patients who need a noncardiac surgical treatment simultaneously. Performing both procedures during a single operation may eliminate unnecessary delay in cancer treatment [5], but was only reported in a few patients. Our cardiothoracic center has performed simultaneous cardiac and noncardiac surgery since 2010. In this study, we analyzed the outcomes of simultaneous coronary artery bypass grafting (CABG) and esophagectomy in a selected group of patients and demonstrated the possibility and feasibility of this simultaneous session.

## Materials and methods

### Patients

This retrospective study was approved by the Ethics Committee of Shanghai East hospital (certificate number: 2017-049). Between September 2010 and August 2016, 22 patients diagnosed with a concomitant heart disease and esophageal or esophageal-gastric junction (EGJ) cancer and requiring surgical treatment in our center were enrolled as group A. Patients who underwent isolated esophagectomy during the same time period were selected as group B for comparison of long-term safety of the combined procedures. The propensity score matching method was used to balance the potential confounders between groups. The two groups were matched using a one-to-two nearest-neighbor matching in terms of age, gender, cardiovascular co-morbidities, and especially the histology (including tumor type and tumor staging) of esophageal cancer. Cancer was staged according to the seventh edition of American Joint Committee on Cancer (AJCC) staging manual [6]. Patients undergoing neoadjuvant therapy were excluded.

The following clinical data were obtained from all patients: age, sex, pathological tumor characteristics, operation time, total estimated blood loss, time of intensive care unit (ICU) stay, time of hospital stay, incidence of complications and recurrence, and survival (Additional file 1). The clinical characteristics of both groups are shown in Table 1. All patients received routine clinical examination, blood serum biochemical examination, electrocardiogram, chest computed tomography (CT), and abdominal ultrasound.

**Table 1:** Clinical characteristic of the patients

Characteristics of patients	Group A	Group B	*P* value
Sample quantity	22	44	
Female (*n*, %)	3 (13.64%)	6 (13.64%)	1.000
Age (years)	65.64 ± 6.67	63.80 ± 6.63	0.293
NYHA class (*n*)			0.000
I	0	43	
II	22	1	
III	0	0	
IV	0	0	
EF (%)	64.09 ± 3.12	65.22 ± 2.95	0.159
CAD classification (*n*)	22	3	0.000
Stable angina (*n*, %)	18 (81.82%)	3 (100.00%)	
Unstable angina (*n*, %)	4 (18.18%)	0	
Number of disease vessels (*n*)	2.05 ± 0.79		
Tumor location			1.000
Esophagus (*n*, %)	13 (59.09%)	26 (59.09%)	
EGJ (*n*, %)	9 (40.91%)	18 (40.91%)	
Hypertension (*n*, %)	12 (54.55%)	15 (34.09%)	0.111
Diabetes mellitus (*n*, %)	4 (18.18%)	4 (9.09%)	0.286
Cerebrovascular disease (*n*, %)	10 (45.45%)	3 (6.82%)	0.000
Smoking (*n*, %)	9 (40.91%)	21 (47.73%)	0.600
Tumor stage (*n*)			0.928
I	6 (27.27%)	13 (29.55%)	
II	7 (31.82%)	12 (27.27%)	
III	9 (40.91%)	19 (43.18%)	

*Abbreviations*: *NYHA* New York Heart Association, *LVEF* left ventricular ejection factor, *CAD* coronary artery disease, *EGJ* esophageal-gastric junction

### Simultaneous operation types

All patients were operated by the same group of surgeons and underwent double-lumen tracheal intubation under general anesthesia. Posterolateral thoracotomy incision was performed along the left sixth or seventh intercostal space according to the tumor location. In group A, off-pump beating-heart coronary artery grafting was operated firstly, followed by esophagectomy. The left internal mammary arteries (LIMA) and/or the saphenous veins were taken as the bypass grafts. Proximal anastomosis was at the descending aorta. Patients in both groups received esophagectomy. Lymph nodes around the esophagus, along the descending aorta and inferior pulmonary ligaments, and below the aortic arch and above the diaphragm were completely dissected. The abdominal lymph nodes were cleared via the transdiaphragmatic approach. The anastomosis was constructed by stapling.

Anticoagulant therapy was done in group A, and low-molecular-weight heparin (LMWH) was given according to body weight and blood loss until the discharge from hospital. Brilinta or Plavix was commenced after LMWH was stopped.

### Follow-up

All patients were followed up in clinic visits: firstly at 1 month after discharge, secondly at 3 months, and then at 6-month interval. All patients received clinical examination, electrocardiogram, cardiac echo, and chest X-rays at each visit.

### Statistical analysis

**Fig. 1:**
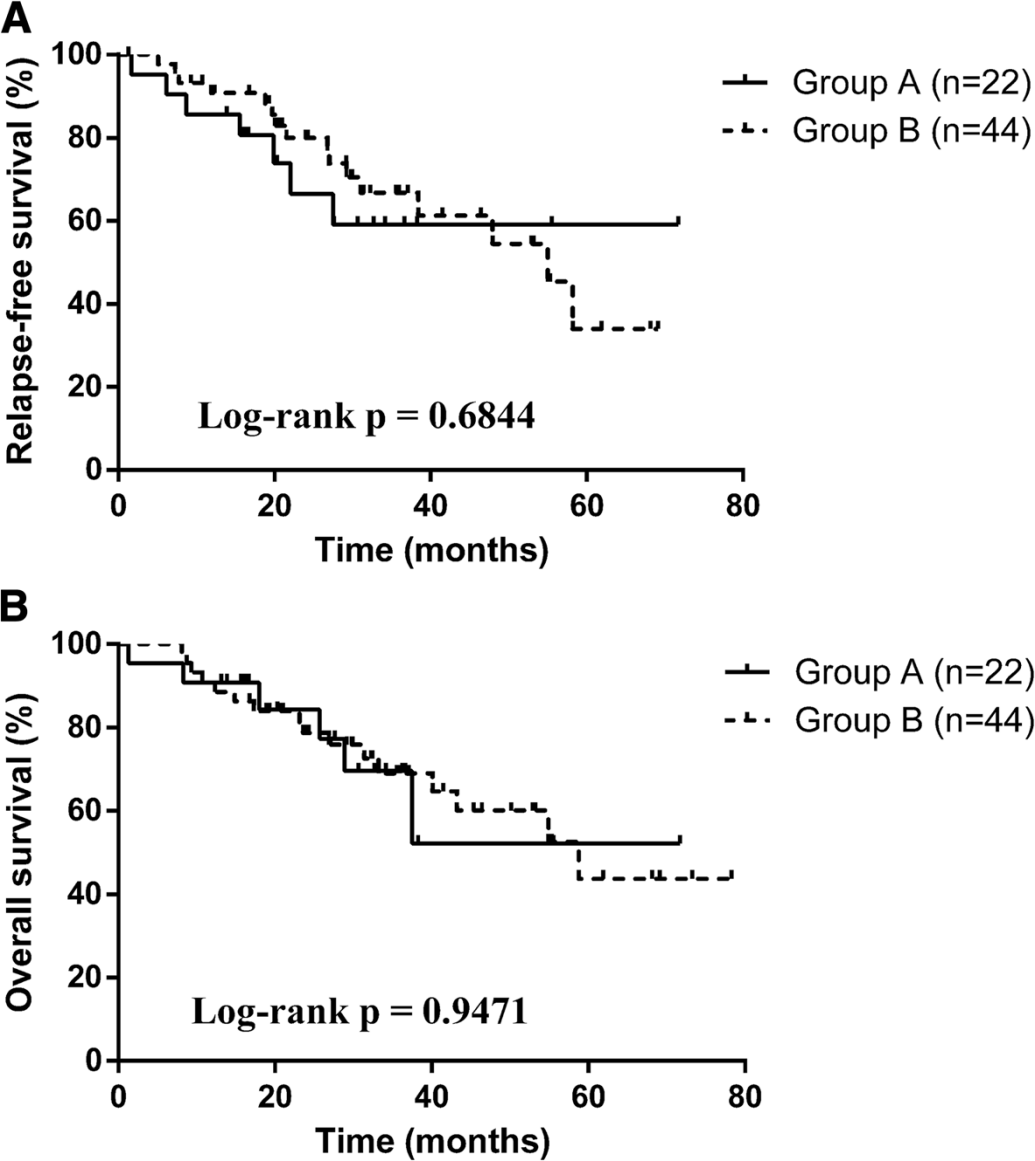
Kaplan–Meier survival curves for relapse-451 free survival (**a**) and 452 overall survival (**b**) according to matched patients in our study

Survival curves were obtained using the Kaplan–Meier method (Fig. 1). Statistical analyses were performed on SPSS 16.0 (SPSS Inc., Chicago, IL, USA). Data were presented as mean ± standard deviation (SD) for continuous variables. Demographic and clinical data between groups were compared via chi-square test or Fisher exact test. *P* < 0.05 was considered to be statistically significant.

## Results

### Patient characteristics

The clinical characteristics of patients are summarized in Table 1. Group A consisted of 19 men and 3 women, while group B involved 6 women and 38 men. The average ages were 65.64 ± 6.67 and 63.80 ± 6.63 years, respectively. As for co-morbidities, remarkable differences between groups were found in diabetes mellitus, hypertension, and smoking as the main risk factors of CHD. As for cardiac function, no significant differences between groups were found in left ventricular ejection fraction (LVEF). The pairing method successfully ranked patients into group B with similar tumor location and staging.

### Surgical outcomes

Surgical outcomes are summarized in Table 2. There was no recurrent myocardial ischemia or death in the perioperative period in either group. The mean operation time and the length of postoperative hospitalization were longer, and the blood loss and postoperative total drainage were more in group A, as a result of two operations performed at one session. Postoperative mechanical ventilation was used in group A, but not in group B. Only one patient in group A was operated by the mid-sternal incision for CABG and the left approach for esophageal cancer. A single incision from left thoracotomy was employed for the remaining patients from both groups. The ICU stay was shorter in group A, but not significantly. The total postoperative hospital stay was significantly longer in group A, considering the greater trauma of the combined surgeries.

**Table 2:** Comparison of surgical outcomes

Variables	Group A	Group B	*P* value
Operation time	404.73 ± 74.22	212.91 ± 48.97	0.000
Blood loss (ml)	606.82 ± 304.84	223.86 ± 122.23	0.000
Surgery plasma transfusion (ml)	312.73 ± 314.25	50.91 ± 116.00	0.000
Red blood cell transfusion (unit)	1.39 ± 1.42	0.38 ± 0.87	0.001
Bypass graft number (*n*)	2.36 ± 1.00	0	/
Mechanical ventilation time (min)	862.27 ± 252.09	0	/
ICU stay (min)	1887.05 ± 931.07	2236.82 ± 4124.66	0.697
Postoperative hospital stay (day)	19.59 ± 6.18	12.77 ± 4.62	0.000
24-h drainage after operation (ml)	216.59 ± 170.11	217.16 ± 155.50	0.989
Postoperative total drainage (ml)	2006.59 ± 976.71	760.91 ± 610.15	0.000
Tumor size (cm)	3.49 ± 1.83	3.08 ± 1.52	0.342
Surgical approach (*n*, %)			0.333
Single incision approach	21 (95.45%)	44 (100%)	
Two-incision approach	1 (4.55%)	0 (0.00%)	
Tumor pathological type			0.236
Adenocarcinoma (*n*, %)	8 (36.37%)	21 (47.73%)	
Squamous cell carcinoma (*n*, %)	10 (45.45%)	21 (47.73%)	
Others (*n*, %)	4 (18.18%)	2 (4.54%)	

*Abbreviations*: *ICU* intensive care unit

Pathological outcomes are summarized in Table 2. All patients were histologically diagnosed. Ten patients were diagnosed as squamous cell carcinoma, 8 as adenocarcinoma, 2 as gland squamous cell carcinoma, 1 as endocrine cell carcinoma, and 1 as small cell carcinoma in group A. Positive lymph node metastasis was identified in 10 (45.5%) of the 22 patients. The postoperative complications, mainly respiratory complications, are minor and curable in both groups (not listed here).

### Follow-up

All the patients from group A were followed up for 1.3 to 71.7 months. Seven patients experienced relapse; finally, five of them died of tumor recurrence or metastasis (8.3, 18.0, 25.7, 28.9, 37.5 months after surgery, respectively), while one patient died of pneumonia after 1.3 months postoperatively, but no patient died of major cardiovascular events during the follow-up. The remaining 16 patients survived for 13.2 to 71.7 months during the follow-up.

All patients in group B were followed up from 8.1 to 78.3 months. Sixteen patients experienced relapse, and finally, 12 patients died. Moreover, other four died of non-cancer-related factors during radio (chemo)-therapy after operation. No patient died of major cardiovascular events during the follow-up. The remaining 28 patients survived from 16.8 to 78.3 months during the follow-up.

The 5-year survival rate was 52.2% in group A and 43.8% in group B (*P* > 0.05).

## Discussion

Our experience shows simultaneous esophagectomy and off-pump CABG can be performed safely and efficiently at a tertiary care center. The path toward the optimal outcome will necessarily take fairly long time, even for operators already skilled in thoracic and cardiac operations. Despite certain differences in clinical indicators between simultaneous operations and single esophagectomy, the simultaneous operations were evidently safe, enabled earlier esophageal cancer resection, and avoided the eventual complications from further surgical procedure.

Esophageal cancer is a leading cause of cancer-related mortality in China [4]. The combination of CHD and malignancy is expected to increase due to an aging population in China and diagnostic technique improvements. Surgery is still the first choice for patients with resectable malignant diseases. The high difficulty in treating cancers accompanied with severe CHD significantly increases operation-related morbidity and mortality [7]. Radiotherapy and chemotherapy are very important cancer treatment techniques, but directly impact the heart. When patients refer to noncardiac operation under general anesthesia, preoperative treatment of CHD is commonly accepted in practice, which may reduce perioperative mortality and morbidity [8].

Cardiac revascularization includes percutaneous coronary intervention (PCI) and CABG. Coronary stents have dramatically improved immediate angiographic results by reducing the incidence of emergent bypass grafting. Consequently, stents are now used in more than 50% of all percutaneous transluminal coronary angioplasty procedures. For patients with coronary stents, intensive anticoagulation treatment greatly increases risk of hemorrhagic complications among those undergoing noncardiac operations. Stent is thrombogenic and requires combined antiplatelet (clopidogrel and aspirin) therapy till endothelialization is completed, which takes 1 month to 1 year according to stent type [9]. During this period, combined antiplatelet therapy must be continued to avoid stent thrombosis. The incidence of fatal perioperative complications is extraordinarily high in patients who undergo noncardiac surgery soon after coronary stent implantation. Hence, when a patient is considered for noncardiac surgery soon after coronary stenting, efforts should be made to avoid PCI if possible [10]. Longer delay in cancer operation may result in cancer progression [11]. As reported, the incidence and mortality of myocardial infarction are lower among patients receiving CABG, compared with the results of noncardiac surgery after either PCI or CABG [10].

Surgical treatment of malignant diseases can be either simultaneously performed with CABG or via a staged approach a few weeks later. However, whether to select either one-stage or two-stage operation remains controversial [12, 13]. A two-stage procedure is two surgical traumas, which may delay the tumor resection and doubles postoperative pains and treatment costs. One method to improve intraoperative and potentially postoperative factors is to utilize a simultaneous surgery for patients with combined cardiac and noncardiac diseases. On the contrary, the one-stage procedure needs double expertise for this complex operation. This procedure first described in 1990 can improve operative time and is at least as safe as conventional surgery. The simultaneous procedure has gradually been adopted to perform various cardiac and noncardiac operations, including pulmonary lobectomy, esophagectomy, gastrectomy, and colectomy [14, 15]. This approach has been mainly performed in thoracic carcinoma patients and may solve two problems through a single incision [12, 15]. However, in China which has a large population, there are few reports of combined esophagectomy and off-pump CABG. By extensively reviewing relevant literature [12, 16], we present the largest single-center report in China so far. The simultaneous surgeries may prolong the anesthesia time and surgical time and increase the postoperative heparinization-related bleeding risks. Nevertheless, the one-stage procedure provides the immediate solution of two intrathoracic operations and minimizes the risk of intraoperative bleeding. Off-pump CABG compared with on-pump coronary revascularization may improve long-term survival and minimize the incidence of bleeding or tumor dissemination secondary to extracorporeal circulation-induced immunosuppression and coagulopathy [15, 17]. Simultaneous off-pump CABG and tumor resection has been performed to treat several cancer diseases [13, 15]. Although our report suggests its safety and efficiency, this combined operation is not suitable for all patients. The patients to undergo this operation should be well selected and prepared in advance. According to our experience, the simultaneous operation indications are (1) Preoperative estimation of esophageal cancer is able to be radical resection, without distant metastasis; (2) Patients suffer from CHD with CABG indication, without emergency operation; (3) Patients should be able to tolerate combined surgery, so patients with EF < 45% or with heart failure are not recommended; (4) Patients having a history of chest or heart operation or with pleural adhesions are not recommended.

The left transthoracic approach performed here is commonly used for middle third or lower third esophageal tumors in China and is outstanding with shorter hospital stay and lower incidence of postoperative complications [18, 19]. Meanwhile, the left lateral thoracotomy incision enables the off-pump CABG. The use of LIMA in CABG graft is considered the best choice for myocardial revascularization. The 10-year patency rate of saphenous vein graft is 40–60%. Here, the CABG grafts were selected depending on the operative incision, malignant degree of cancer, coronary artery conditions, and life expectancy. The saphenous vein graft can supply blood to heart muscles and help to avoid cardiovascular events during the life expectancy, when the internal mammary artery cannot be easily harvested as a graft. In group A, 4 patients underwent internal mammary arteries grafting and 18 patients underwent saphenous vein grafting, and the mean number of anastomosed coronary vessels was 2.36.

Esophageal cancer is extremely aggressive, as its 5-year survival rate is about 23–46% [20, 21]. Because of the high mortality, all efforts should be made to limit operation-related mortality and morbidity of esophageal cancer. Since surgeon-related factors can contribute to morbidity [22, 23], all operations in our study were conducted by the same team of surgeons. In our center, we performed almost ten thousand esophagectomy or three thousand off-pump CABG in recent decades, which proved our experience and skills were particularly important in achieving good outcomes of combined operations. These surgeons had both thoracic and cardiac skills, which is rare under the current highly professional background. In addition, the preoperative patient selection and the postoperative professional care and nursing also guaranteed patient recovery. After the thoracic operations we have accomplished so far, few patients have complications following esophageal surgery. Patients from both groups had no recurrent myocardial ischemia or death during the perioperative period. Patients from group A had longer mean operation time and more blood loss, as a result of the two operations performed at one session. Only one patient in group A was operated by the mid-sternal incision for CABG and the left approach for esophageal cancer, because his condition was worsened during the surgery and two separated incisions would shorten the revascularization time. The remaining 21 patients were operated by one surgical incision. The 5-year survival rates and relapse-free survival rates were both similar between groups, indicating cardiac surgeries add no risk of complications or mortality to those patients undergoing surgical treatment of malignancy.

This study has several limitations. Firstly, it is a retrospective study. Secondly, the sample size is relatively small, and it is difficult to reach significant power when accessing the results between groups. Thirdly, the oncological outcomes of this series are not confirmed by long-term study, which should be done in the future. Finally, the technical modifications are in part positively influenced by the ongoing gained experience.

Despite these limitations, we believe this is a useful work in guiding surgeons who want to establish a simultaneous approach at their institutions. Our experiences hopefully may provide them some advice when they consider this simultaneous operation for the first time.

In conclusion, the simultaneous approach can be performed effectively and safely by experienced surgeons and is safe and beneficial for the selected group of patients. Sub-optimal outcomes may occur perioperatively, which can be solved through dedication, experience, and progressive technical development. The off-pump CABG adds no negative effect on the life expectancy of patients.

## Conclusions

The risk of simultaneous esophagectomy and coronary artery surgery is not high. Despite the certain differences in clinical indicators between groups, the simultaneous procedures are evidently safe, enable earlier esophagectomy, and can avoid the eventual complications from further surgical procedure.

## Additional file

Additional file 1: Clinical data of two groups. (XLSX 19 kb)

## Abbreviations

AJCC: American Joint Committee on Cancer
CABG: Coronary artery bypass grafting
CHD: Coronary heart disease
CT: Computed tomography
EGJ: Esophageal-gastric junction
ICU: Intensive care unit
LIMA: Left internal mammary arteries
LMWH: Low-molecular-weight heparin
LVEF: Left ventricular ejection fraction
PCI: Percutaneous coronary intervention
SD: Standard deviation
